# Medical and Physical *Intelligence*

**Published:** 1799-06

**Authors:** 


### MEDICAL AND PHYSICAL
### *INTELLIGENCE,*
### (Original and Selected.)

THE tenth Volume of the Transactions of the French Royal Society of Medicine, and the fifth Volume of the Prize Questions of the Academy of Surgery, have been lately published by the professors of the School of Medicine at Paris, in three volumes, quarto. This extensive and multifarious '*Extract*' contains, as may be expected, a great number of articles equally interesting and useful to medical science. From these we shall extract some particulars of *Vicq-d'Azyr's Eloge*, of *Poulletier de la Salle*, whose first work was a Translation of the London Pharmacopoea, to which he added a Dictionary of the Materia Medica. Vicq-d'Azyr, in treating of the difficulty and importance of a complete medical code, introduces reflections which will be the more interesting to our readers, as they relate to the progress of practical medicine, perhaps now as far distant from perfection, as it was at the time they were written.

"The members of the London College of Physicians, uniformly remark, and it cannot be too often repeated, that there is no greater obstacle to the progress of the medical art, than the practice of mixing a great number of drugs, in the same formula. Thus we are ignorant to which of the remedies the success is due; and unless the particulars are ascertained, our judgment must ever remain uncertain.

"Besides, if notwithstanding the labours of the most learned societies, we have not yet a good pharmacopoea, it proceeds only from the difficulty of the task. To mix in the old compositions substances truly active, and to retain them; to search in the writings and practice of the most eminent physicians, what remedies are most successful, and the improvements that can be made on them; to consult even empiricism, and profit by its daring experiments in judging by observation, rather than submitting to the theories of the day; to reject the numerous compounds which are employed without any just cause, as they are compounded without reason; to impart to medicines a uniform strength, the result of which may be calculated; to bring them to a state of simplicity, which can leave no doubt of their effects; and never to deviate from the rules prescribed by chemistry; that is to say to unite to a perfect knowledge of the history of medicine a profound study of its auxilary sciences; to acute feeling, a correct understanding; to the greatest prudence, that boldness which is necessary to the attainment of any end—such are the talents and qualities required in those who undertake to regulate our formulæ.

"Already have the discoveries of Bergmann and Scheele produced in this respect a beneficial revolution. The physicians of Stockholm first set the example. Those of Wirtemberg, Geneva, Edinburgh, and London followed. The Medical Faculty at Paris is at this time engaged in the same reform. May we not hope, that all the colleges, impelled by the progress of of knowledge, may erase from their dispensatories informal recipes, monstrous assemblages of contradictory substances, whose virtues are counteracted, and which have been sanctioned only by ignorance? The numerous votaries of ancient superstition boast of their brilliant æras; they assert that we have only pulled down, without rebuilding; that we have substituted nothing for what we have destroyed. Vain declamation! too often repeated by the opponents of science.

"The moment the clouds are dispelled, the sun succeeds; and truth, like light, is one of the most valuable presents that can be made to human nature.

"The authors of the London Pharmacopœa having spoken freely against Fernel, one of the most illustrious physicians of the French School, Poulletier complains loudly of that jealousy."

"Poulletier had begun a great number of works which he did not complete. Among these sketches is to be distinguished an Essay on the accidents produced by the admission of air into the different cavities of the human body. From his researches on this subject, it is established that the small intestines are more irritable than the great ones, and that the uppermost parts of the latter possess a greater degree of irritability than the other parts."

Among the other manuscripts of Poulletier de la Salle, of which Cit. G. Roi is the depositary, are to be found the details of experiments on Sensibility; remarks on the means of dissolving the extremity of a leaden probe in the bladder; but the most remarkable tract in this collection, is a number of observations on congestions of the brain. From this work and the inquiries of Walter, is established the great medical truth, that a number of acute diseases terminate in a sort of apoplexy, in which the brain, surcharged with moisture, loses its activity, and sinks under the oppression.

Poulletier is likewise the author of enquiries on soda and kali; and of an analysis of bones, which afforded him, what Gahn and Scheele had merely announced, phosphoric acid and lime. Indefatigable in his other labours, and animated by the same excessive desire of discovering new truths, he examined sedative salt and bile; in the latter of which he discovered spermaceti, which has since been found in a great mass in the cemetery of the Innocents, by the commissioners of the Society of Medicine, who superintend the digging up of the bodies.

Poulletier has also analyzed Farina, and commenced, in conjunction with Fourcroy, in 1786, a series of experiments on the human calculus.

The other physicians, to whose manes Vicq d'Azyr offers a tribute of praise in this volume, are Bourdois de la Mothe, Thion de la Chaume, le Houx, Duvernin, Dupuy, Destrapieres, Dosan, and Manneti, associates and correspondents of the Society of Medicine.

---

Among the articles contained in the extracts lately published from the memoirs of the School of Medicine, we find two very ingenious essays, the one by Citizen Jurine, and the other by Citizen Gattoni, with observations thereon, by Citizen Seguin, on the following important question:

"What advantages can medicine derive from the modern discoveries in the art of ascertaining the purity of the air, by the different eudiometers?

Citizen Jurine's memoir which gained the prize, in 1787, is divided into four parts:

The first relates to the modification of atmospherical air by respiration.

The second treats of the air which is exhaled by the skin, and of the nature of ambient air in different diseases.

The

The third explains the difference of atmofpherical air, the air of hofpitals, and fick rooms.

We fhall examine each of thefe fections of Cit. Jurine's work fucceffively, taking notice at the fame time of the principal obfervations made by Seguin.

In the firft place, Jurine remarks on the opinions, the views, and the fimple difcoveries of Prieftley, the Abbé Fontana, Scheele, Bergmann, and Morozzo, and the folid and luminous theory of Lavoifier and La Place. He next explains the extent and capacity of the lungs, after Hales and Jurin. Afterwards he analyzes the air exfpired in different circumftances of life and health.

The means of analyzation were lime water, nitrous gas, and the flame of a wax candle.

Experiments were in the firft place made to afcertain the difference relative to age, fex, the ftate of fafting, and the effect of digeftion. The air produced by the moft complete exfpiration was then divided into four parts, and fucceffively fubmitted to a comparative analyfis. The other experiments were intended to afcertain the changes produced in the air exfpired in fever, the laft ftage of confumption, blood-letting, refpiration in pure oxygen, or in air rendered lefs refpirable from previous refpiration Citizen Hallé having already publifhed a part of the refults of thefe experiments, under the article *Air* in the *Dictionnaire de Médec. de l'Encycl: Meth:* it is unneceffary to repeat them.

To this firft part of Cit. Jurine's memoir, Cit Seguin directs his principal objections, which directly attack the accuracy of the eudiometers, the lime water, and nitrous gas, employed.

The other obfervations relate to the quantity of carbonic acid gas formed during refpiration, and the pretended formation of another gas.

In the fecond part, Cit Jurine explains feveral experiments made upon himfelf, the refult of which were, that he difengaged a certain quantity of carbonic acid gas by the fkin. Cit. Seguin thinks, on the contrary, that the carbonic gas does not iffue through the fkin, but is only that formed by the act of refpiration, which, defcending by its fpecific weight, in the bath where the Genevefe phyfiologift made his experiments, had difplaced the atmofpheric air from the veffels he employed.

Citizen Hallé has again offered the principal facts deduced from the experiments contained in the third part. The objection of Citizen Seguin againft their refult in general, that there is uniformly produced a kind of *mephites* in the inteftinal canal, is, that, on the contrary, the predominance of azote in the large inteftines, according to the experiments of Jurine, ought not to be attributed to the atmofpheric air contained in the ftomach and inteftines lofing a portion of oxygen, and the azotic gas becoming, by that means, more abundant.

The fourth part of this work exhibits a fhort view of the improvements medicine has received from pneumatic chemiftry, which, in the report, is brought down to the prefent period.

The memoir of M. Gattoni, like the preceding, is intended to give a folution of the phyfiological problem propofed by the Medical Society, and contains the particulars of a variety of refearches and experiments. The author, after relating the hiftory of the nitro-aërial eudiometer, and fhewing the incorrectnefs of that inftrument, and its general infufficiency, to afcertain the change of the air by the different kinds of miafmata, adopts the eudiometer of Volta, which he defcribes. The experiments made with this latter inftrument, like thofe made with all the others, have only elucidated the different different proportions of azote, oxygen, and carbonic acid gas varioufly modified, by different airs, or by the ftate of the patients on whom the experiments were tried, or by the different caufes of difeafe.

---

In a memoir by Citizen Delunel, read before the Medical Society of Paris, the 22d Pluviôfe (February 10, 1799), upon opium, and the different preparations of its extract, which alfo contains an inquiry, whether the refin of opium may be ufeful in medicine, we find a new procefs for preparing the extract, which, with the author's obfervations thereon, we fhall give in his own words,

" It is eafy", fays Delunel, " to adminifter opium in a dry form, either in powder or pills; but its effects are, perhaps, to be dreaded, on account of the difficulty of diffolving it in fimple watery fluids, or in thofe which it may meet in the ftomach. The difficulty confifts in liquifying it, without employing as a folvent alkohol or any other fpirituous liquor. For this purpofe I have employed Rouffeau's procefs, namely, fermentation; with this difference, that the refin of the opium was employed in place of the opium entire. Six ounces of the refin of opium, one pound and a half of honey, and fix pounds of water, were expofed together to the fun for fix weeks; the fermentation was vifible at the end of eight days. When it was over, and the liquor filtered, I found fix drachms of refin diffolved in it. I left this mixture for a month, to difcover if there actually was formed a combination of the refin of the opium. A fpontaneous evaporation with a near approach, in confiftence, to extract, the addition of potafs and ammonia to the beforementioned folution, have evinced the prefence of the refin of opium. I fhall make only one remark, that the fulphuric acid does not act here in the fame way as with the juice or extract of feveral vegetables, that is to fay, it produces no precipitation. In confidering the procefs which I have defcribed, the folution of the refin of the opium may perhaps be attributed to a fpirituous principle, formed during the fermentation by the help of the honey. To fatisfy myfelf upon this point, I diftilled a part of my product; and the liquor diftilled contained fo little of the principle of alkohol, that I found it impoffible to diffolve, by its means, any quantity of refin, and the refiduum of the diftillation has always remained clear, with a very ftrong putrid odour: whence I conclude, that the divifion of the refin of the opium in water is as much owing to its combination with other principles, as with that of alkohol which has hitherto been believed to be its only folvent.

" I have made the fame remark as Baumé, in his 'Pharmacopœa', when fpeaking of Rouffeau's drops, namely, that there is formed on the furface of the liquor a pellice, which he attributes to its mouldinefs, while I confider it to proceed from the purification of the honey and of the refin itfelf. The filtration takes away as much as is required, and I have not feen that the produce was the refin itfelf."

---

M. Abich, a German chemift, has lately publifhed in '*Crell's Annals of Chemiftry*', a chemical inquiry into the nature and properties of the *honey-ftone*. This fubftance is found in Thuringia, between beds of bituminous wood, and is fometimes near an inch in diameter; it is cryftallized in great maffes, in a double pyramidal form, with four fides; it bears a great refemblance to amber, but does not, like that fubftance, become electric by rubbing; its fpecific gravity does not exceed 1.666,

It further appears from M. Abich's analyfis, that fifty parts of this foffil are compofed of eight parts of the carbonate of alum, two of carbon, one one and a half of oxyd of iron, twenty of carbonic acid, fourteen of the water of cryftalization emitting the fmell of bitter almonds, and two and three-quarters of ether. Total forty-eight, one fourth.—The lofs of one and three-fourths, and the agreeable odour which was exaled during the operation, the exact nature of which the author had not been able to afcertain, determined him to repeat his experiments with fome variation. The fecond analyfis added to the produce of the firft, a fmall portion of the benzoic acid.

An analyfis of the fame ftone made by M. Lampadius, afforded the following conftituent parts, viz. 86.40 of carbon, 3.50 of petroleum, 2.00 of filex, and 3.00 of the water of cryftallization. Hence Von Crell is of opinion, that thefe two chemifts have examined different ftones.

In confequence of the refult of *his* experiments, and on account of the perfect incombuftibility of the honey-ftone, M. Abich propofes to exclude it henceforth from the clafs of combuftible bodies, and to place it among that of aluminous earths.

---

The *Academy of Surgery*, at *Copenhagen*, have recently made an important change in the examinations for degrees. The candidates are now fhut up in the anatomical theatre, where they diffect particular parts of a fubject, and reduce the refult of their obfervations to writing, inftead of giving them *vivâ voce.*

---

As feveral eminent writers have denied the poffibility of *diftinct impregnation*, it may be ufeful to collect the facts that feem to favour its exiftence.

In the '*Recueil Periodique*,' No. xxvi. we meet with the following curious account of twins, one a perfect birth, and the other apparently of fix months only; communicated by Roch Tarbes, officer of health at Toulofe:

"In the month of September, 1790, the wife of Cit. Noel, about thirty years of age, and much inclined to corpulency, was in the laft month of pregnancy. She had irregular pains for eight days; on the ninth fhe fell in labour, and I delivered her of a boy in the natural way.

"As I put my hand into the uterus, to get at the placenta, I difcovered in the vagina the feet of a fecond child, enveloped in its own feparate membranes: which, being broken, produced a difcharge of water, and afterwards a female child, that was delivered by the feet with the greateft facility.

"The two placentæ were perfectly diftinct, having each feparate membranes. The moft remarkable circumftance was, the difproportion fubfifting between the two children; the boy being eighteen, while the girl was only twelve inches high; fhe had no nails, and was not better formed than a child in the fixth month of pregnancy. As fhe could not fuck, fhe was kept alive eight days with cow's milk."

This cafe is attefted by *Noel, the father*, now furgeon in ordinary, of the fecond clafs, to the Military Hofpital of Touloufe.

---

The following Prize Queftions have been propofed by the Medical Society of Paris, for the prefent year:—

1. "To determine the nature of the lymph; its ufe in the animal economy, and the advantage which medicine has derived, and may ftill derive, from the modern difcoveries refpecting the ftructure and functions of the lymphatic fyftem.

2. "To determine, by experiments and obfervations, what are the medicinal properties of phofphorus, and the phofphoric and phofphorous acids; in what difeafes they may be employed with advantage, and in what manner they act.

3. "To determine, from anatomical inquiries, and inductions drawn from practice, what is the caufe of aneurifm, according to the nature of the difeafe, its ftate. the part which it affects, and the circumftances attending it.

4. "To determine, by accurate experiments, the influence of oxygen in the animal economy, particularly in the treatment of difeafes, both internal and external.

5. "To explain the caufes and fymptoms of *tetanus traumaticus;* to afcertain their diverfity, and manner of cure.

6. "Are there any precurfory fymptoms of the tetanus or trifmus traumaticus?"

---

Profeffor Schranck, of Ingolftadt, has lately enriched the fcience of botany with a work treating on that very curious and interefting branch, called *the phyfiology of vegetables.* The author has taken a particular view of the fecondary veffels of plants, by which he underftands the down, the fpiculæ, the glands, and the veficles. His principal object is to fhew, that thefe veffels may afford an additional mean of determining with accuracy, the claffification of Linnæus, when his diftinctions are equivocal; and that fuch veffels, according to *Malpighi,* form the extremities of thofe canals in which the fluids, that fupply the plant with nourifhment, circulate; and which only inhale the air without exhaling it.—Much has been done in this curious branch of botanical fcience, by the ingenious labours of *Hales, Ingenhouz, Duhamel, Bonnet,* and *Sennebier,* but the German Profeffor aims at a fyftematic arrangement of this fafcinating fubject, which has not been attempted by his predeceffors.

---

Citizen Du Mont Courset has, in a letter to the Editor of the '*Magafin Encyclopedique*', made fome very ingenious obfervations on an opinion of *Van Helmont* and *Boyle,* quoted by *Boiffonarde,* "that plants draw their principal nourifhment from water." Some account of thefe obfervations will be given in our next Number.

---

It is acknowledged by all botanifts, that an "herbarium vivum" is an excellent mean of promoting the ftudy of this ufeful fcience; and that fuch a work is particularly wanted to illuftrate the complicated and extenfive clafs of the *Cryptogamia.*

Mr. J. A. Hose, of Heidelberg, has devoted much time and attention to inveftigate the nature of moffes, fo that he has at length publifhed the firft Number, containing twelve plates, or twelve different fpecies, of his "*Herbarium vivum Mufcorum frondoforum cum defcriptionibus analyticis ad normam Hedwigii.*" This elegant collection is accompanied with copious explanations in Latin and German, which muft render it an acceptable prefent to the Englifh ftudent defirous of acquiring fome knowledge of the German. Upon the whole, it may juftly be called a fplendid little performance; and we have only to regret that the author cannot furnifh more than two fuch Numbers every year.

---

Cit. Delile, who is now in Egypt, in the capacity of botanift of the expedition to that country, has made feveral important difcoveries of plants. As we propofe to give a more particular account of Delile's botanical exploits, in a future Number, we fhall at prefent mention only the difcovery of a new fpecies of palm-tree, to which he has given the name of '*Le Palmier Doum*', and which has not been hitherto fatisfactorily defcribed.

---

It

In our last Number we promised to communicate to our readers two chemical processes lately invented by Dr. Richter, The first is an impro ved and profitable method of preparing the *crystallized acid of lemons*, from the juice of either fresh or putrid lemons, currants, &c. and deserves the preference over every other hitherto contrived, because when the juices, which is generally the case, are saturated with calcareous earth, the citrat of lime thus produced, retains a part of the extracted matter which cannot be separated by ablution, and which consequently unites with the acid, and renders the crystallization difficult. The acid juice, therefore, should be saturated in a boiling heat with vegetable alkali, the exact quantity of which should be particularly attended to. After this add such a proportion of the muriat of lime, as can be decomposed by the vegetable alkali before employed. This proportion may be ascertained either by previous experiment. or from the table shewing the capacities of the elective attraction of bodies, inserted in Dr. Richter's fifth Number of his "*Reflections on the Modern Subjects of Chemistry*, While the mixture before specified, is boiling for a quarter of an hour, the muriatic and citric acids exchange their combinations; the former produces the muriat of potass, and the latter is precipitated in the form of citrat of lime. This, after being completely edulcorated, must be dried, and afterwards digested in such a quantity of diluted sulphuric acid, as is required for saturating the calcareous earth contained in the citrat of lime: the point of saturation ought either to be determined by previous experiment, or regulated by Dr. Richter's tables of decomposition given in the work before mentioned.

After having properly managed this part of the process, the separated citric acid must be evaporated in a similar manner as is done with the acid of tartar; the sulphat of lime still settling must be carefully separated; the acid is then to be mixed with water in which carbon is diffused, and after the mixture is perfectly clear and pellucid, it is exposed to a slow, spontaneous evaporation, which will produce beautiful crystals of the size of peas. From twenty-six quarts (Silesian measure, nearly the same as English) of the juice of putrid lemons, Dr. Richter obtained upwards of a pound of crystallized acid.—See *Crell's Annals of Chemistry*. Vol. II. p. 380.

---

The *Citrat of Iron*, to the discovery of which Dr. Richter was led in his stochiometric experiments, deserves a distinguished place among our officinal chalybeates. And although few physicians on the Continent have hitherto prescribed it as a medicinal remedy, yet we are entitled to say upon the authority of Dr. Frank, of Gnesen, that he has found this compound of singular benefit in many diseases.

It is, in every respect, a neutral salt, the preparation of which Dr. Richter performs in the manner following:—Equal parts of iron-filings reduced to powder, and crystallized acid of lemons, or the residuum which is obtained after the crystallization of the citric acid, are mixed; a sufficient quantity of water is then added, and the mixture stirred for some time. Afterwards it is exposed either to the heat of the sun, or kept in some warm place, till it be gradually inspissated, during which time, however, it ought to be frequently stirred up. Of the dry mass a lixivium must be made with water, which dissolves a considerable part of it. This solution, if evaporated to dryness, affords the citrat of iron in the form of a light brown powder, which is readily soluble in water. The residuum of the preceding preparation, if mixed with more water, slowly inspissated, then reduced to a lixivium, and evaporated again to dryness, produces repeatedly the same result, without any further addition of citric acid, till at last the iron iron and lime in a combined ftate remain behind, without poffeffing any acid. *Ibid.* p. 382.

---

Dr. Valerian Aloys Brera, Profeffor of Medicine, in the Univerfity of Pavia, has lately been under the neceffity of refigning his chair, on account of the political intrigues and party factions that have begun to difturb that ancient feat of the mufes. Dr. Rasori, a young man, and a more enthufiaftic Brunonian, has been appointed his fucceffor. This change however, we are happy to add, will not affect Profeffor Brera's literary publications (Vid. No. II. p. 203 of our Journal); on the contrary, we are authorifed to fay that he will continue his exertions with increafed zeal. Vid. *Jena Intelligenzblatt.* No. XXIX. p. 388.

---

Among the late *Inaugural Differtations* which characterife the *Leipzig College*, we meet with two deferving of particular notice. One of thefe was defended by Dr. H. W. Richter, and entitled: "*Medici ex omni parte perfecti imago*;" the other is a continuation of Vice Chancellor Dr. Hasse's Differtation, (Sect. II.) *De iis quae* (we would rather fay *qui*) *artem difficilem reddunt.*

---

Englifh literature, and particularly medical literature, is much valued on the Continent, if we may form fome comparative judgment from the tranflations of Englifh works into the German language, which are continually advertifed in the Literary Gazette of Jena. There appears to be a fpirit of rivalfhip prevailing among both tranflators and bookfellers, who generally conclude their advertifements with faying, that they have obtained an early copy of the work from England, (fometimes even before it is publifhed here) and thus claim a right of priority in publifhing a German tranflation. Such is the cafe with the following three works which have been announced in the Intelligence-papers annexed to the Jena Gazette, by feveral bookfellers, now contending for the prior right of publication; "viz. *Haflam, on Infanity; Home, on Ulcers of the Legs*; and *Rollo, on the Diabetes Mellitus.*" *Vide Jena Intelligenzblatt:* No. L. for April. 1799. p. 399.

---

We noticed in our firft Number, p. 86, the extenfive practice, importance, and utility of Mr. Collier's *new machines for percolation*, (applicable to all fluids, but more particularly WATER) for which he had obtained a Patent: we have now much fatisfaction in adding, that on farther enquiry, we highly approve of thefe inventions, grounded on the only true principles that can fhorten this procefs, which has hitherto been always found tedious in the extreme.

Firft, he removes the difficulty refulting from the clear fluid paffing through its own impurities, by taking a direction from the external parts of the filtering medium inwards, from which it is drawn off.

Secondly, an urged preffure is employed, which fpeedily forces it through the percolating pores.

Thirdly, though the apparatus takes up comparatively no room, and the expence is very eafy, it is fo contrived as to contain more filtering furface in contact with the fluid, than a large ftone does when full.

The method Mr. Collier has employed to deprive *any* quantity of fluid of its colour, or putrefcent qualities, by a given quantity of charcoal, is likewife highly ingenious.

Mr. SHERWIN, of Enfield, intends to publish a work on the " Affections both morbid and salutary, excited in the human frame by external absorption."—In the year 1787, Mr. Sherwin communicated a paper on this subject to the Medical Society of London, a part of which, relating to the absorption of emetic tartar, was published in the second Volume of their Memoirs. In the introduction to that paper, which was not published by the Society, appears the following passage, which recent discoveries respecting the cow-pox render striking :

" May it not be conceived, that some particular unsuspected substance, applied to the human frame, in a state of moisture or effluvia, may be imbibed, and excite an infectious disorder, which shall afterwards propagate itself from one patient to another? Small-pox, measles, infectious sore throat, &c. may have been thus originally excited, though mankind may for ever be ignorant of their respective sources. "

---

We learn that Mr. PARKINSON, is about to enlarge his work of "*Medical Admonitions*" by the addition of a table, pointing out the degrees of danger manifested by various symptoms; and an Essay on the injurious consequences of the excessive indulgence of children.—Such a work is truly a desideratum in English literature, and cannot fail to be productive of the most beneficial effects, especially among those classes of society, which are prejudiced in favour of quackery.

---

At a quarterly meeting of the Royal College of Physicians, of Edinburgh, held on the 7th of May, Dr. GEORGE GAVIN BROWNE, Physician in Bath, and Dr. ALEXANDER WILSON, Physician in Montrose, were elected Fellows of the College; and Dr. ROBERT KENNEDY, Physician in Edinburgh, was admitted a Licentiate.

---

Dr. BRADLEY, having been requested to postpone his summer-course of *Lectures on the Theory and Practice of Medicine*, till after the Hospital Lectures were concluded, proposes to commence on *Monday* the tenth of *June*, at the Lecture-room, No. 21, Great Eastcheap, near the Monument, at six in the evening.

The Course will consist of about seventy lectures.—Terms, three guineas.

---

Two works of Travels, both possessing much curious and original information, are now in the press and will speedily be published. One is that of Mr. BROWNE, the African traveller, who has visited the interior parts of that extensive continent, which have hitherto been considered as *terra incognita*. We have seen a considerable part of this work, and can speak from conviction of its great merit, both with respect to novelty, and the useful information it contains.—The next publication is that by the celebrated naturalist and philologer, Dr. PALLAS, of St Petersburgh, whom the late Empress, with her usual munificence and liberality, sent to explore the *Southern Provinces of the Russian Empire*, in the years 1793 and 1794. These travels, the first volume of which is just published at Leipzig, will be comprised in two volumes, quarto, and embellished with near one hundred coloured plates and maps, illustrative of the manners, dress, and customs of the various Tartar nations, and of different subjects relative to the *Natural History* and *Antiquities* of a tract of country extending to several thousand miles in length, and never before described.—A faithful translation of this splendid work, from the original German into the English language, will shortly appear, by *Dr. Willich*, and *Mr. S. Porter*, of Trinity College, Cambridge.

Cambridge.—The Englifh edition will (perhaps the firft inftance of the kind, in this country) be ornamented with all the plates contained in the German, and re-engraved by the *original artift*, *Mr. Geisfler*, of Leipzig, who accompanied Dr. Pallas on his travels, and made alfo the drawings and defigns.

---

A work which reflects credit on the author, as well as the artift by whom the plates are executed, deferves particular notice in our Journal; and we have now to mention one which will, when completed, be the moft comprehenfive, correct, and practical fyftem of anatomy ever attempted in this or any other country. We allude to the "*Syftem of Diffections*," by Mr. Charles Bell, of Edinburgh (in folio); the third part of which is juft publifhed. It contains the diffections of the peritoneum; inferences drawn from views of the parts; the fection of the pelvis; points of furgery illuftrated by that fection; the contents of the pelvis as feen from behind, and a plan of the arteries; of the defcent of the tefticle; of hernia, hydrocele, &c.—The fourth number of this elegant work, we underftand, has already left the prefs at Edinburgh, and is on its way to London, where the work is publifhing by Johnfon, Callow, and other medical bookfellers.

---

In our firft number, p. 86, we mentioned the eftablifhment of an American Mineralogical Society, in the city of New-York: we are happy to announce a fimilar inftitution in this country, which promifes to be of fingular advantage.—Mr. *W. H. Pepis*, of the Poultry, London, has lately circulated printed propofals, in the character of Secretary to "The British Mineralogical Society," from which we extract the following particulars: "They will analyze, *free of expence*, package and carriage excepted, for the proprietors of mines or landed eftates, whatever fubftance they may meet with, in fufficient quantity, to render a knowledge of the component parts a defirable object. In return, the Society expects that fingular or curious fpecimens of the mineral productions, which may be met with in working the Britifh mines, will be fent for the improvement of the Society's Cabinet, which will thus in time become a national ornament.

"When the analyfis of any mineral fent for examination is completed, the refult, with an account of the methods employed, will be fent to the proprietor of the fpecimen; and if it fhall contain any new fubftance, the probable ufes to which it may be applied, will be fuggefted."

---

Profeffor Blumenbach, of Goettingen, in his "Obfervations on the bodily and mental capacity of Negroes," *Magazin für das Neuefte aus der Phyfik*, Vol. IV., ftates, that having had an opportunity of feeing and converfing with many negroes, and procured for his collection a great many anatomical preparations from negro bodies, he is from thence, and what he has read in different voyages, convinced of the truth of the following propofitions: 1. That between one negro and another there is as much (if not more) difference in the colour, and particularly in the lineaments of the face, as between many real negroes, and other varieties of the human fpecies; and 2, That negroes, in general, in regard to their mental faculties and capacity, are not inferior to the reft of the human race.

---

The fifth volume of the "*Memoirs of the Medical Society of London*" is, we underftand, nearly printed; it is expected to appear in the beginning of next month.

